# Ether lipid deficiency disrupts lipid homeostasis leading to ferroptosis sensitivity

**DOI:** 10.1371/journal.pgen.1010436

**Published:** 2022-09-30

**Authors:** Marcos A. Perez, Andrea J. Clostio, Isabel R. Houston, Jimena Ruiz, Leslie Magtanong, Scott J. Dixon, Jennifer L. Watts

**Affiliations:** 1 School of Molecular Biosciences and Center for Reproductive Biology Washington State University, Pullman, Washington, United States of America; 2 Department of Biology, Stanford University, Stanford, California, United States of America; Worcester Polytechnic Institute, UNITED STATES

## Abstract

Ferroptosis is an iron-dependent form of regulated cell death associated with uncontrolled membrane lipid peroxidation and destruction. Previously, we showed that dietary dihomo-gamma-linolenic acid (DGLA; 20: 3(n-6)) triggers ferroptosis in the germ cells of the model organism, *Caenorhabditis elegans*. We also demonstrated that ether lipid-deficient mutant strains are sensitive to DGLA-induced ferroptosis, suggesting a protective role for ether lipids. The vinyl ether bond unique to plasmalogen lipids has been hypothesized to function as an antioxidant, but this has not been tested in animal models. In this study, we used *C*. *elegans* mutants to test the hypothesis that the vinyl ether bond in plasmalogens acts as an antioxidant to protect against germ cell ferroptosis as well as to protect from whole-body tert-butyl hydroperoxide (TBHP)-induced oxidative stress. We found no role for plasmalogens in either process. Instead, we demonstrate that ether lipid-deficiency disrupts lipid homeostasis in *C*. *elegans*, leading to altered ratios of saturated and monounsaturated fatty acid (MUFA) content in cellular membranes. We demonstrate that ferroptosis sensitivity in both wild type and ether-lipid deficient mutants can be rescued in several ways that change the relative abundance of saturated fats, MUFAs and specific polyunsaturated fatty acids (PUFAs). Specifically, we reduced ferroptosis sensitivity by (1) using mutant strains unable to synthesize DGLA, (2) using a strain carrying a gain-of-function mutation in the transcriptional mediator MDT-15, or (3) by dietary supplementation of MUFAs. Furthermore, our studies reveal important differences in how dietary lipids influence germ cell ferroptosis versus whole-body peroxide-induced oxidative stress. These studies highlight a potentially beneficial role for endogenous and dietary MUFAs in the prevention of ferroptosis.

## Author summary

Ferroptosis is a regulated form of cell death driven by excess production of lipid peroxides. Understanding ferroptosis is important because this type of cell death is associated with disease states such as neurodegeneration and various renal, liver, and lung diseases. In addition, with more knowledge of regulatory mechanisms, induction of ferroptosis could be harnessed to control tumor growth. To examine specific lipid contributions to ferroptosis sensitivity, we used mutant strains of the small roundworm *Caenorhabditis elegans* exhibiting a range of altered lipid compositions to examine how cellular and dietary lipids influence ferroptosis and oxidative stress sensitivity. We found that the accumulation of lipid peroxides correlates with cell death but that plasmalogens, a subset of ether lipids that are proposed to act as cellular antioxidants, do not protect from ferroptosis or oxidative stress. Instead, ether lipid biosynthesis contributes to lipid homeostasis in membranes. In their absence, membrane saturated fatty acids are increased, while membrane monounsaturated fatty acids (MUFAs) are less abundant. Restoring membrane MUFAs reduces lipid peroxides and contributes to cellular resistance to ferroptotic cell death. These studies suggest that dietary MUFAs could prevent ferroptosis in disease.

## Introduction

Ferroptosis is a regulated form of cell death characterized by iron-dependent accumulation of lipid peroxides and subsequent cellular destruction [1–3]. Early studies of ferroptosis focused on the regulation of antioxidant systems, such as the availability of reduced glutathione, the regulation of glutathione peroxidase enzymes, and the actions of radical trapping antioxidants, in the protection against ferroptosis [1–7]. However, the identification of oxidized arachidonic acid (AA; 20:4(n-6)) and adrenic acid (AdA; 22:4(n-6)) in cells undergoing ferroptosis unveiled the importance of lipid peroxidation in the promotion and execution of ferroptotic cell death [8–11]. Since then, other groups have shown a range of polyunsaturated fatty acids (PUFAs) may drive this process, and identified important roles for membrane remodeling enzymes, such as acyl-CoA synthetase (ACSL) 3 and 4 and lysophosphatidylcholine acyltransferase 3 (LPCAT3) in ferroptosis regulation [9, 12–15]. Glycerophospholipids, neutral lipids, sphingolipids and ceramides, cholesterol, and more recently ether glycerophospholipids have all been implicated in ferroptosis [3, 8, 9, 13, 16–20]. How these different lipids contribute to ferroptosis is poorly understood.

**Fig 1:**
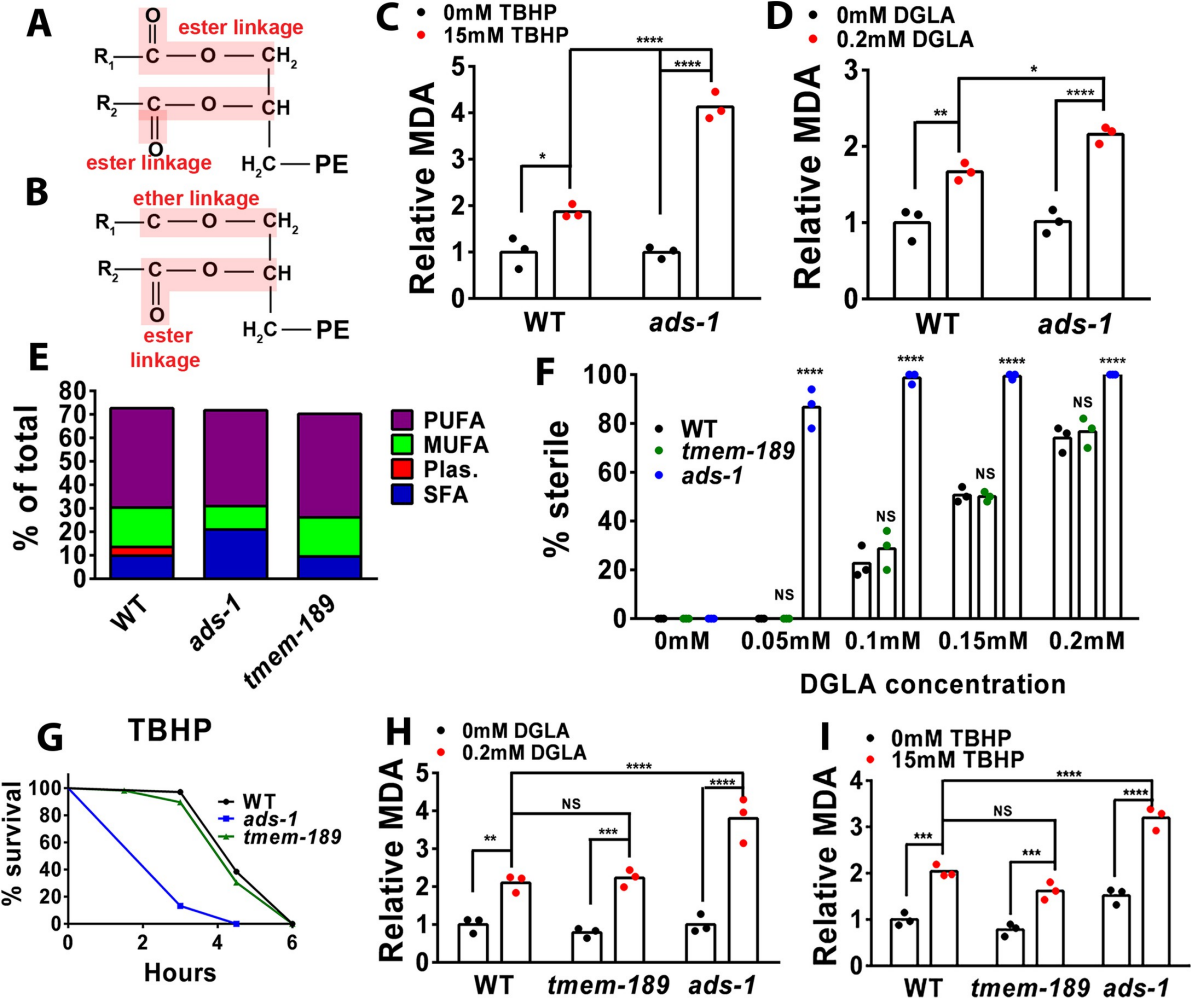
Plasmalogens are not required for protection in DGLA-induced ferroptosis or peroxide-induced oxidative stress. (A and B) structures of a typical phospholipid with two ester-linked fatty acids (A) or an “ether lipid”, with one ether linked fatty acid and one ester linked fatty acid (B). (C,D, H and I) Young adult worms were harvested and the end-product of lipid peroxidation, malondialdehyde (MDA), was measured and normalized to total protein using BCA. Each data point represents an independent experiment of 1,250 worms for each treatment. Statistical significance was determined using a two-way ANOVA with Tukey’s test for multiple comparisons summarized in S2 Table. TBHP, tert-butyl hydroperoxide; DGLA, dihommo-gamma-linolenic acid. (E) Simplified fatty acid composition in wild type, *ads-1*, and *tmem-189*. Detailed fatty acid composition of each strain with averages and standard deviations in S1 Table. PUFA, polyunsaturated fatty acid; MUFA, monounsaturated fatty acid; Plas, plasmalogen; SFA, saturated fatty acid. (F) Percentage (%) sterility in wild type, *ads-1*, and *tmem-189* raised on 0.05mM, 0.1mM, 0.15mM, and 0.2mM DGLA. Each data point represents an independent experiment of 50 worms for each treatment. Statistical significance was determined using a two-way ANOVA with Tukey’s test for multiple comparisons. Shown are the comparison of the mutant vs. WT, all comparisons are summarized in S2 Table. (G) Oxidative stress survival assays were performed with 14.7 mM TBHP. Wild type and *ads-1* were used as controls to compare with the survival of *tmem-189*. Approximately 100–200 worms were used for each strain per treatment. Statistical significance for survival was determined using log rank tests (Mantel Cox) and summarized in S2 Table. Fatty acid composition and sterility for (E, F and H) are displayed in S1 Table. Statistical differences shown are NS, not significant, * P<0.05, **P<0.01, ***P<0.001, ****P<0.0001.

Ether glycerophospholipids (herein simplified to ether lipids) contain a fatty alcohol attached in the sn-1 position in the glycerophospholipid backbone through an ether bond, instead of a conventional ester linkage (Fig 1A and 1B). There are two different types of ether lipids: the alkyl O-linked ether lipids, and alkenyl vinyl-linked ether lipids, canonically known as “plasmalogens”. Both start as fatty alcohols that are incorporated into the precursors of ether lipids in the peroxisomes, then are transported to the endoplasmic reticulum for completion of lipid synthesis and, for a subset of ether lipids, conversion to plasmalogens by the addition of an alkenyl double bond by the enzyme TMEM189, also known as PEDS1 [21–23]. Both classes of ether lipids are important structural components of membranes, act as signaling molecules, and are implicated in different peroxisomal diseases, neurodegenerative disease, and cancer [21, 24–26]. There is evidence from liposome and cell culture experiments that plasmalogens protect against reactive oxygen species (ROS) [27, 28]. The vinyl ether bond has been proposed to act as an antioxidant, because when free radicals such as ROS and lipid peroxides attack the vinyl double bond, an innocuous aldehyde is generated, acting as a peroxide “trap” and thereby inhibiting the propagation of lipid peroxidation products [28, 29]. However, this antioxidant model has not been tested in vivo.

Much like ferroptosis, lipid peroxidation is also implicated in general oxidative stress. Studies in *Caenorhabditis elegans* and other organisms use tert-butyl hydroperoxide (TBHP), a toxicant that generates cytosolic reactive oxygen species (ROS) and induces non-specific lipid peroxidation, to study oxidative stress [30–32]. In *C*. *elegans*, TBHP exposure leads to reduced survival from the accumulation of lipid peroxides and increased transcriptional response of oxidative stress response pathway genes, such as the glutathione-S-transferase *gst-4* [31, 33–35].

Previously, we showed that ingestion of the omega-6 PUFA dihomo-gamma linolenic acid (DGLA; 20:3(n-6)) led to ferroptotic cell death of germ cells in *C*. *elegans* and in human cancer cells [13]. *C*. *elegans* strains carrying a mutation in the *ads-1* gene, encoding alkyglycerone phosphate synthase (AGPS), are completely deficient in ether lipid biosynthesis [36], and were more sensitive to DGLA-induced ferroptosis (Perez et al., 2020). These studies supported a role for ether-linked lipids in protection from ferroptosis. Similarly, we previously showed that ether lipid-deficient mutant strains are also more sensitive to whole-body oxidative stress after exposure to TBHP and accelerated death [36]. Thus, both stress and death modalities point to a common protective mechanism by ether lipids in the context of lipid peroxidation. However, the role of ether lipids in ferroptosis regulation remains controversial, especially in mammalian cells [19, 20, 37].

In this study, we used *C*. *elegans* to examine how ether lipids and unsaturated fatty acids impacted DGLA-induced ferroptosis and TBHP-induced oxidative stress. We found that even though ether lipid biosynthesis is protective from DGLA-induced ferroptosis, this protection does not arise from the vinyl double bond in plasmalogens. Instead, modulation of stress resistance by ether lipids is strongly dependent on endogenous MUFA and PUFA synthesis, and specific dietary and endogenous PUFAs play different roles in ferroptosis versus peroxide-induced oxidative stress. We discovered a role for the mediator complex MDT-15 in both DGLA-induced ferroptosis and peroxide-induced oxidative stress in the absence of ether lipids and suggest that this protection is mediated through regulation of endogenous MUFA biosynthesis. Our findings shed light on the differences in the reported effects of ether lipid biosynthesis in the promotion or protection of ferroptosis and suggest that the presence of endogenous omega-6 PUFAs, not ether lipids per se, is the main driver of ferroptosis. Finally, as has been shown in mammals, our work emphasizes that dietary MUFAs strongly protect from ferroptosis.

## Results

### Ether lipid deficiency leads to higher lipid peroxidation end products

Our previous studies showed that ether lipid biosynthesis is required to protect *C*. *elegans* from two types of oxidative damage. Ether-lipid defective mutants showed greatly increased sensitivity to DGLA-induced germ cell ferroptosis [13] as well as accelerated death after exposure to the toxicant TBHP [36]. To determine if the presence or absence of ether lipids influenced oxidized lipid levels in both ferroptosis and peroxide-induced oxidative stress, we measured the levels of malondialdehyde (MDA), a common end product of lipid peroxidation [38]. Wild type worms and ether-lipid deficient *ads-1* worms were treated with either DGLA or TBHP, and MDA levels were compared to untreated controls. Although basal levels of MDA are similar in wild type and *ads-1* mutants, the *ads-*1 mutants accumulated higher MDA levels after exposure to both TBHP and DGLA (Fig 1C and 1D), consistent with a role for ether lipids acting to reduce lipid peroxidation in cells.

### Plasmalogens do not protect from DGLA-induced ferroptosis or whole-body oxidative stress

*C*. *elegans* ether lipids consist of approximately one third plasmalogens (vinyl ether bonds) and two thirds O-linked ether lipids [36]. Plasmalogens are synthesized from O-linked ether lipids in organisms ranging from bacteria to humans by the addition of the double bond at the sn-1 position by the TMEM189 enzyme [22, 23]. If the protective effects of ether lipids in ferroptosis and peroxide-induced oxidative stress are due to the vinyl ether bond and the subsequent prevention of lipid peroxide propagation, then we expected that a knockout of *tmem-189* would lead to increased sensitivity to DGLA-induced ferroptosis and TBHP-induced oxidative stress. We obtained a CRISPR deletion mutant strain *tmem-189(syb2649)* and analyzed fatty acid composition using gas chromatography-mass spectrometry (GC-MS). This strain showed a complete absence of 18:0 plasmalogen, indicating that the *C*. *elegans* TMEM-189 homolog shares corresponding plasmanylethanolamine activity that has been shown in bacteria and mammals (Fig 1E and S1 Table). Interestingly, aside from the absence of the 18:0 plasmalogen, these mutants showed relatively normal levels of saturated, monounsaturated (MUFA) and polyunsaturated fatty acids (PUFAs) compared to wild type, in contrast to the ether-lipid deficient mutant *ads-1*, which contain much higher levels of saturated fatty acids and lower levels of MUFAs than wild type (Fig 1E and S1 Table). To test if plasmalogens are playing a role in protection from DGLA-induced ferroptosis, we assessed germ cell loss in wild-type and *tmem-189* mutant animals exposed to various doses of DGLA. Surprisingly, *tmem-189* mutants displayed no difference in sterility levels compared to wild type worms, while *ads-1* mutants had completely sterile populations (Fig 1F). We also examined whether peroxide-induced oxidative stress was influenced by plasmalogens by scoring survival on TBHP. Again, much like in DGLA-induced ferroptosis, *tmem-189* mutants had survival rates that were comparable to wild type worms on TBHP, while the *ads-1* mutants died much faster (Fig 1G). We then used the MDA assay to quantify lipid peroxidation levels in WT, *tmem-189* and *ads-1* strains after exposure to DGLA or TBHP. We found that after treating *tmem-189* mutants with either DGLA or TBHP, levels of MDA were similar to WT, but *ads-1* consistently displayed higher MDA levels (Fig 1H and 1I). Collectively, these data show that while ether lipids are important for protection from DGLA-induced ferroptosis and TBHP-induced oxidative stress, this protection is not due to the plasmalogen subclass of ether lipids, and therefore does not support the “trap” theory of oxidative stress protection by plasmalogens.

### Differential promotion of ferroptotic germ cell death and whole-body oxidative stress by endogenously synthesized C20 PUFAs

**Fig 2:**
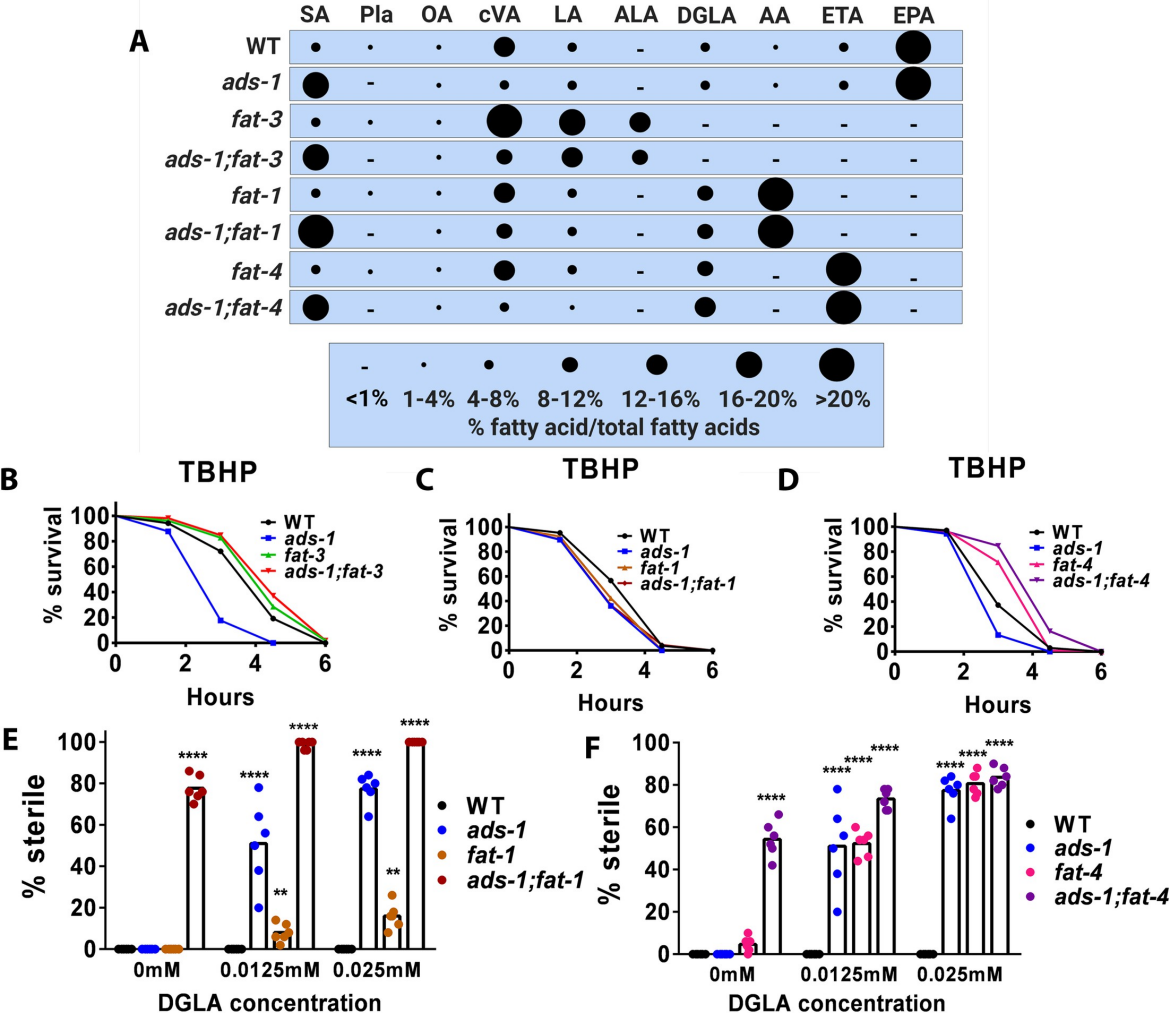
Endogenously synthesized PUFAs differentially modulate ferroptosis and peroxide-induced oxidative stress. (A) Relative fatty acid composition of strains used for this study determined using gas chromatography-mass spectrometry. Complete composition of each strain with averages and standard deviations are displayed in S1 Table. SA-stearic acid (18:0), Pla-plasmalogen, OA-oleic acid (18:1(n-9)), cVA-cis-vaccenic acid (18:1(n-7)), LA-linoleic acid (18:2(n-6)), ALA-alpha linolenic acid (18:3(n-3)), GLA-gamma linolenic acid (18:3(n-6)), STA-stearidonic acid (18:(4n-3)), DGLA-dihomo-gamma linolenic acid (20:(3n-6)), AA-arachidonic acid (20:(4n-6)), ETA-eicosatetraenoeic acid (20:(4n-3)), EPA-eicosapentaenoic acid (20:(5n-3)). (B,C, and D) Survival of young adult wild type, *ads-1*, *fat-3*, and *ads-1;fat-3* on 14.7mM tert-buryl hyderoperoxide (TBHP) ((E, F) Percentage (%) sterility in young adult worms of the indicated genotype raised on DGLA In (B, C, and D) approximately 100–200 worms were used for each strain per treatment. Statistical significance for survival was determined using log rank tests (Mantel Cox) and is shown in S2 Table. In (E and F), each dot represents an independent experiment of 50 worms for each treatment. Statistical significance was determined using a two-way ANOVA with Tukey’s test for multiple comparisons shown in S2 Table. For (E and F), fatty acid composition and sterility data are reported in S1 Table. Statistical differences compared to WT are * P<0.05, **P<0.01, ***P<0.001, ****P<0.0001.

A compelling finding from our previous studies was that ether lipid biosynthesis was completely dispensable for protection against DGLA-induced ferroptosis of germ cells in the absence of endogenously synthesized PUFAs. Specifically, *fat-3* mutants, which cannot synthesize C20-PUFAs, along with *ads-1;fat-3* double mutants, were resistant to DGLA-induced ferroptosis (Fig 2A and S1 Table) [13, 39, 40]. We asked whether these double mutants were resistant to peroxide-induced oxidative stress as seen in *ads-1* mutants. To test this, we placed *fat-3* and *ads-1;fat-3* mutants onto TBHP supplemented plates. Similar to the ferroptosis assays, we found that *fat-3* and *ads-1;fat-3* survived longer than wild type worms on TBHP, even though *ads-1* single mutants died much faster (Fig 2B). These data show that in both DGLA-induced ferroptosis and in whole-body oxidative stress, endogenously synthesized long-chain PUFAs exacerbate the severity of oxidative stress, especially in the absence of ether lipids.

Our previous studies showed that *C*. *elegans* mutant strains with altered PUFA content were either more sensitive or more resistant to sterility induced by dietary DGLA, depending on the amount of endogenously synthesized DGLA in the strains [39]. Two of the sensitive strains were knockouts that eliminated either FAT-1 (omega-3 desaturase) or FAT-4 (Delta-5 desaturase). Both mutant strains accumulate high levels of DGLA, but the *fat-1* mutants also accumulate high levels of AA, while the *fat-4* mutants lack AA and accumulate the unusual omega-3 fatty acid eicosatetraenoic acid (ETA; 20:4(n-3)) (Fig 2A and S1 Table). Both strains lack eicosapentaenoic acid (EPA; 20:5(n-3)), the most abundant C20 PUFA in wild type *C*. *elegans* (Fig 2A and S1 Table). We hypothesized that mutants deficient in both ether lipid biosynthesis and accumulating excess omega-6 PUFAs, including DGLA and AA, would be hyper- sensitized to TBHP-induced whole-body oxidative stress and DGLA-induced germ cell ferroptosis. We reasoned that increased, long chain oxidizable omega-6 PUFAs that drive ferroptosis, as demonstrated in germ cell death via DGLA in *ads-1* mutants, might also sensitize ether lipid deficient mutants with high omega-6 PUFAs in TBHP-induced whole-body oxidative stress [13, 36, 41]. To test this hypothesis, we generated the *ads-1;fat-1* and *ads-1;fat-4* double mutants and induced peroxide stress. For TBHP-induced whole-body oxidative stress, we found that compared to the WT control, the *fat-1*, *ads-1*, and *ads-1;fat-1* double mutants all were more sensitive than WT (Fig 2C, P<0.02 for all mutants compared to wild type, S2 Table). However, the *ads-1;fat-4* strain showed opposite results, with *fat-4* and *ads-1;fat-4* worms showing increased resistance to the TBHP-induced oxidative stress than WT (Fig 2D).

This finding shows an important difference between TBHP-induced stress and germ cell ferroptosis due to DGLA. Interestingly, without adding any additional DGLA, we observed sterility and germ cell loss in the *ads-1;fat-1* strain. After adding dietary DGLA to these strains, we found that these double mutant animals were very sensitive to low levels of dietary DGLA (Fig 2E). Since the *fat-1* worms were fertile in unsupplemented conditions, it is apparent that the presence of ether lipids was protective in the context of excess endogenously synthesized omega-6 PUFAs. Similarly, the *ads-1;fat-4* double mutants were also sterile on media that did not contain dietary DGLA and they were very sensitive to low levels of dietary DGLA (Fig 2F). Overall, these data indicate that accumulation of excess DGLA in mutant strains is not detrimental in the context of TBHP-induced oxidative stress, but points to a role for AA and EPA in promoting and accelerating the effects of TBHP.

### Differential promotion of ferroptotic germ cell death and peroxide-induced whole-body oxidative stress by dietary C20 PUFAs

**Fig 3:**
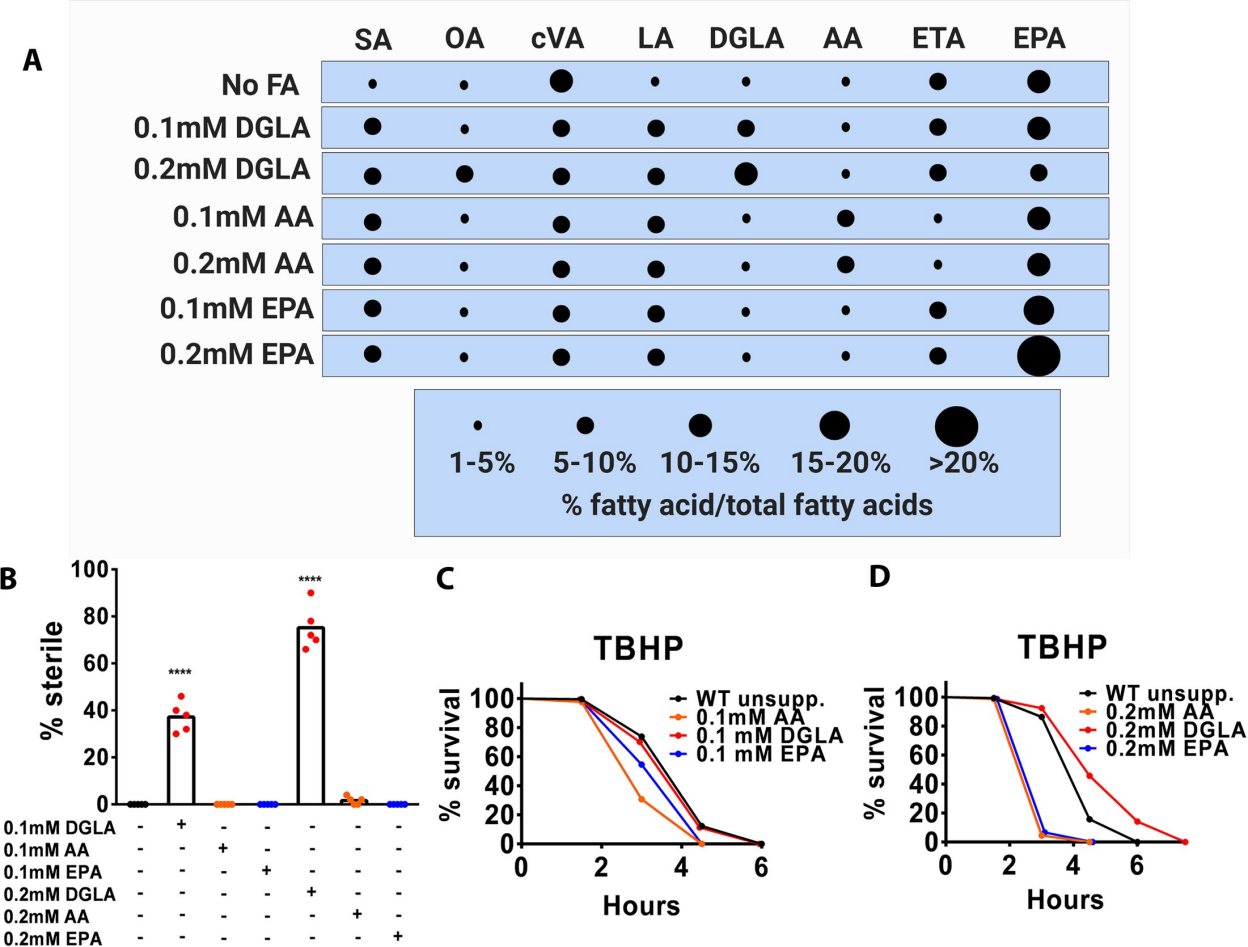
Exogenous 20-carbon PUFAs differentially modulates ferroptosis and peroxide-induced oxidative stress. (A) Relative fatty acid composition in wild type worms treated with DGLA, AA, and EPA determined using gas chromatography-mass spectrometry. SA-stearic acid (18:0), Pla-plasmalogen, OA-oleic acid (18:1(n-9)), cVA-cis-vaccenic acid (18:1(n-7)), LA-linoleic acid (18:2(n-6)), ALA-alpha linolenic acid (18:3(n-3)), GLA-gamma linolenic acid (18:3(n-6)), STA-stearidonic acid (18:(4n-3)), DGLA-dihomo-gamma linolenic acid (20:(3n-6)), AA-arachidonic acid (20:(4n-6)), ETA-eicosatetraenoeic acid (20:(4n-3)), EPA-eicosapentaenoic acid (20:(5n-3)) (B) Percentage (%) sterility of wild type wormsraised on the indicated fatty acids. (C and D) Survival of young adult worms raised on the indicated fatty acids before exposure to 14.7mM tert-butyl hydroperoxide (TBHP). In (B) each dot represents an independent experiment of 50 worms for each treatment. Statistical significance was determined using a two-way ANOVA with Tukey’s test for multiple comparisons summarized in S2 Table. In (C and D) approximately 100–200 worms were used for each strain per treatment. Statistical significance for survival was determined using log rank tests (Mantel Cox) is shown in S2 Table. In (B, C and D), fatty acid compositions are reported in S1 Table.

Whether DGLA and TBHP induce the same form of cell death was unclear at the start of our studies. Understanding these differences is important as TBHP is commonly used to model general oxidative stress but may not accurately model the ferroptosis phenotype observed in mammalian cells and other systems. Since the *ads-1;fat-1* and *ads-1;fat-4* double mutant strains showed different results in the TBHP survival studies and the germ cell ferroptosis assays, we verified the specificity of C20 PUFAs in both assays by supplementing WT worms with exogenous DGLA, AA, and EPA (Fig 3A and S1 Table). We found that at similar doses of these PUFAs, only dietary DGLA promoted ferroptotic germ cell death (Fig 3B). However, when supplementing WT worms with these PUFAs and placing them on TBHP-supplemented media, remarkably, the worms treated with 0.1mM DGLA survived as well as WT (Fig 3C) and survived longer when treated with 0.2mM DGLA (Fig 3C and 3D). On the other hand, AA and EPA pre-treated worms died much faster than unsupplemented worms in a dose-dependent manner (Fig 3C and 3D). Thus, these data clearly demonstrate a difference between ferroptosis and TBHP-induced whole-body oxidative stress, because ferroptotic germ cell death is triggered specifically by DGLA, while whole-body oxidative stress is accelerated by AA and EPA, which are the most unsaturated PUFAs found in *C*. *elegans*.

### The mediator component, MDT-15, protects from ferroptotic germ cell death and peroxide-induced whole-body oxidative stress in the absence of ether lipids

The global genetic regulation of oxidative cell death sensitivity is poorly understood. We sought to link the observed changes in ferroptosis and oxidative stress sensitivity to specific transcriptional regulators. An important feature in the *ads-1* mutants besides the lack of ether-linked lipids is the significant change in fatty acid composition, especially the increase in the saturated fatty acid, stearic acid (SA; 18:0), and the depletion of MUFAs [36]. We previously found that strains carrying loss of function mutations in regulators of fatty acid desaturation, including *nhr-49*, *nhr-80*, and *sbp-1* were very sensitive to DGLA-induced ferroptosis [42]. To examine fatty acid desaturation in the context of ether lipids, we made use of mutations in the transcriptional mediator complex component MDT-15/MED15, for which both gain of function and loss of function mutations are available.

MDT-15 is an evolutionarily conserved co-activator in eukaryotic transcriptional regulation of fatty acid desaturation, and in stress-response [35, 43, 44]. In *C*. *elegans*, knockdown and loss-of-function *mdt-15* mutant strains display increased SFAs, and reduced MUFAs and PUFAs [43, 45]. Furthermore, transcriptional analyses convey that they not only have reduced fatty acid desaturase expression, but of reduced stress response-related genes as well and are very sensitive to TBHP stress [35, 43, 45]. Recently, *mdt-15* gain-of-function mutants have been generated and found to increase membrane fluidity and protect from SFA toxicity from high glucose diet and toxic metal stress [46–50].

**Fig 4:**
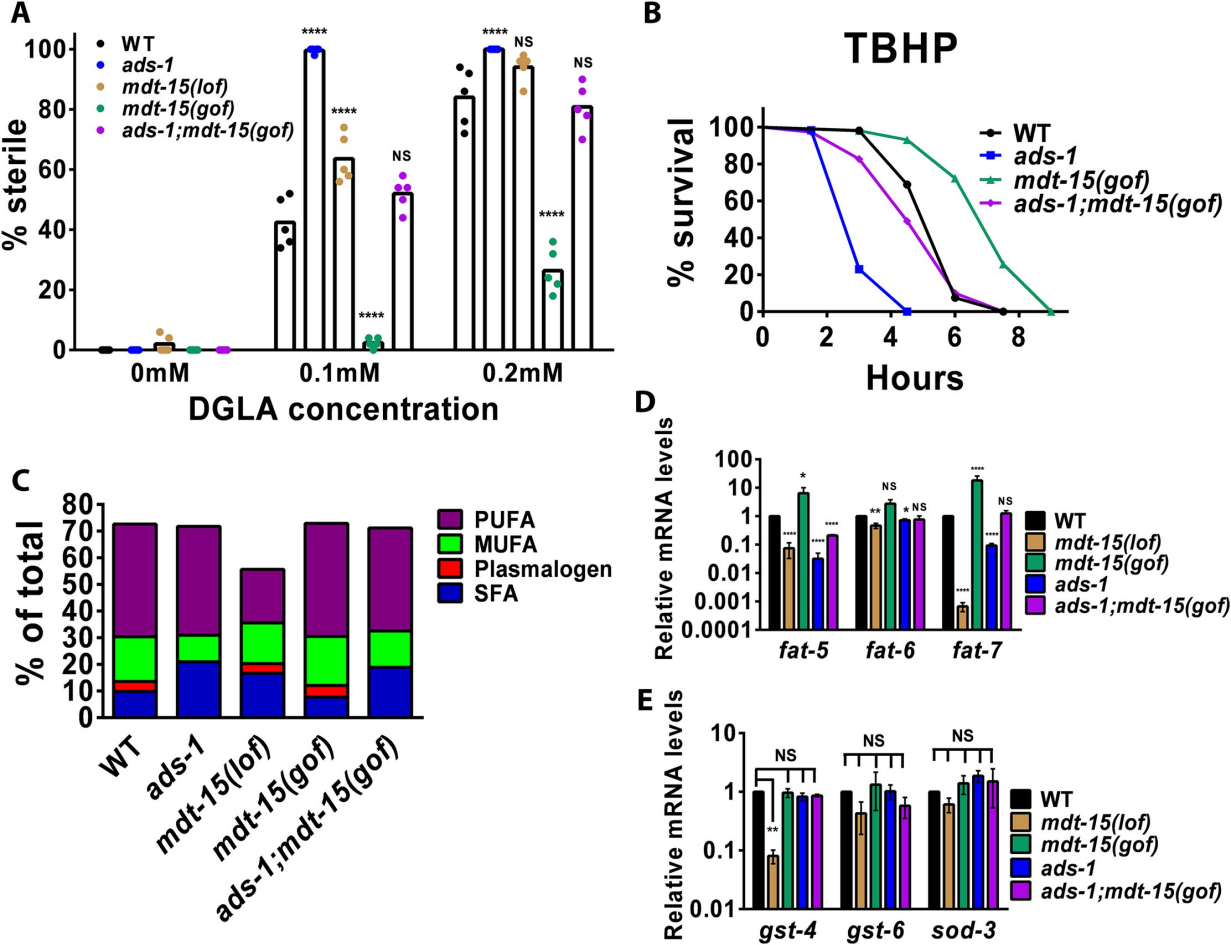
The mediator complex, MDT-15, plays a protective function through increased expression of Delta-9 desaturases in ether lipid deficiency. (A) Percentage (%) sterility in young adult worms of the indicated genotype raised on dihommo-gamma linolenic acid (DGLA). (B) Survival of young adult worms exposed to 14.7mM tert-butyl-hydroperoxide (TBHP). (C) Relative fatty acid composition determined with gas chromatography-mass spectrometry of strains used in (A) and (B). (D and E) Fold-change of basal mRNA levels in mutant worms relative to wild type worms grown on standard nematode growth media. mRNA levels were normalized to *cdc-42* and Y45F10D.4. In (A) each dot represents an independent experiment of 50 worms for each treatment. Statistical significance was determined using a two-way ANOVA with Tukey’s test for multiple comparisons summarized in S2 Table. In (B) approximately 100–200 worms were used for each strain per treatment. Statistical significance for survival was determined using log rank tests (Mantel Cox) and is shown in S2 Table. In (C), values do not add up to 100% because dietary cyclopropane fatty acids and several others are not displayed in this chart. The complete fatty acid composition and sterility data are reported in S1 Table. In (D and E), student’s t-tests were performed to determine statistical significance and summarized in S2 Table. Statistical differences compared to WT are NS, not significant, * P<0.05, **P<0.01, ***P<0.001, ****P<0.0001.

Given these findings, we asked if the *mdt-15(et14)* gain-of-function (herein referred to as *mdt-15(gof)*) could ameliorate the severity of survival and germ cell ferroptosis of *ads-1* mutants after exposure to TBHP and DGLA. We generated the *mdt-15(gof)*;*ads-1* double mutant strain and compared it to WT and *mdt-15(tm2182)* (herein *mdt-15(lof)*). We found that the *mdt-15(lof)* strain was highly susceptible to DGLA-induced ferroptosis, while the *mdt-15(gof)* were resistant (Fig 4A). Interestingly, *ads-1;mdt-15(gof)* displayed ferroptotic germ cell death comparable to WT (Fig 4A), suggesting that even in the absence of ether lipids, the *mdt-15(gof)* aided in a return to homeostasis in the *ads-1* background. Since the *mdt-15(lof)* were previously shown to be sensitive to TBHP stress, we next placed the *mdt-15(gof)* and *ads-1;mdt-15(gof)* on TBHP supplemented plates. The *mdt-15(gof)* strikingly survived much longer than WT on TBHP (Fig 4B). *ads-1;mdt-15* also survived much longer than *ads-1*, but had a similar return to homeostasis when compared to WT worms (Fig 4B). Overall, these data suggest that *mdt-15(gof)* returns the ether-lipid deficient *ads-1* mutants to WT levels of oxidative stress protection from DGLA-induced ferroptosis or in peroxide-induced whole-body oxidative stress assays.

We next examined the lipid composition of these strains and found that compared to *ads-1*, there was a modest reduction in overall SFA in *ads-1;mdt-15(gof)* (18.9%) compared to *ads-1* (20.9%) while the MUFA composition increased to 13.7% in *ads-1;mdt(gof)* versus 10.0% in *ads-1* (Fig 4C and S1 Table). This shows that compared to *ads-1*, *ads-1;mdt-15(gof)* lipid compositional changes are influenced by enhanced activity of MDT-15. Since MDT-15 is a transcriptional co-activator of fatty acid desaturation as well as genes involved in oxidative stress response, we asked if *mdt-15(gof)* is influencing fatty acid desaturation or oxidative stress responses in *ads-1* mutants on a transcriptional level. We measured the basal levels of transcription of three genes involved in oxidative stress response (*gst-4*, *gst-6*, and *sod-3*) and three genes involved in the conversion of saturated fatty acids to MUFAs (*fat-5*, *fat-6*, and *fat-7*). We found that *mdt-15(lof)* and *ads-1* had lower expression of *fat-5* and *fat-7*, while *mdt-15(gof)* showed higher expression of the same genes (Fig 4D). When assessing oxidative stress response-related genes, *gst-4*, *gst-6*, and *sod-3*, we found that *mdt-15(lof)* was the only strain to have a significantly lower expression of these three genes compared to WT, and all other strains were not significantly changed (Fig 4E). Overall, these data suggest that transcription regulation of MUFA synthesis in *ads-1;mdt-15(gof)* is playing a key role in protection from DGLA-induced ferroptosis and peroxide-induced whole-body oxidative stress.

### Endogenous and dietary MUFAs protect from DGLA-induced ferroptosis sensitivity

**Fig 5:**
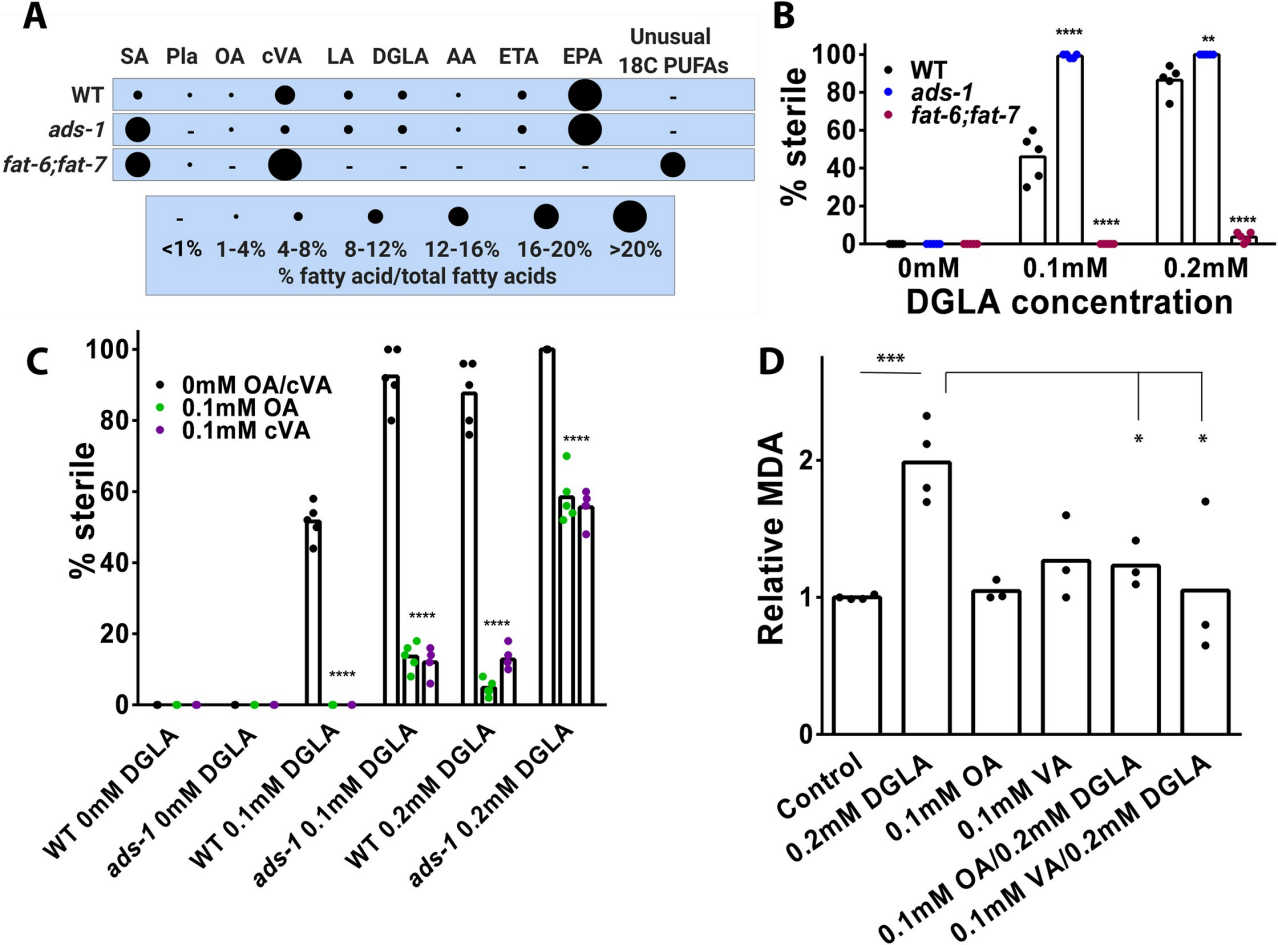
Dietary MUFAs are protective in DGLA-induced ferroptosis through inhibition of lipid peroxidation. (A) Relative fatty acid composition of worms of the indicated genotype as determined with gas chromatography-mass spectrometry. SA-stearic acid (18:0), Pla-plasmalogen, OA-oleic acid (18:1(n-9)), cVA-cis-vaccenic acid (18:1(n-7)), LA-linoleic acid (18:2(n-6)), ALA-alpha linolenic acid (18:3(n-3)), GLA-gamma linolenic acid (18:3(n-6)), STA-stearidonic acid (18:(4n-3)), DGLA-dihomo-gamma linolenic acid (20:(3n-6)), AA-arachidonic acid (20:(4n-6)), ETA-eicosatetraenoeic acid (20:(4n-3)), EPA-eicosapentaenoic acid (20:(5n-3)) (B and C) Percentage (%) sterility in young adult worms of the indicated genotype raised on the indicated fatty acids. In (B and C) each dot represents an independent experiment of 50 worms for each treatment. Statistical significance was determined using a two-way ANOVA with Tukey’s test for multiple comparisons summarized in S2 Table. Fatty acid composition and sterility for (B, C, and D) are reported in S1 Table. In (D) each data point represents an independent experiment of 1,250 worms for each treatment. Statistical analysis was performed with Student’s t-test and P values are reported in S2 Table. * P<0.05, **P<0.01, ***P<0.001, ****P<0.0001.

We and others previously showed that dietary MUFAs are protective in ferroptotic cell death [12, 13]. We previously showed that *fat-2* mutants encoding the delta-12 desaturase, which accumulate OA and lack C20 PUFAs, were highly resistant to DGLA-induced ferroptosis. Similarly, co-treatment of DGLA with OA protected germ cells in both WT and *ads-1* worms [13]. Another gene that is regulated by MDT-15 is *fat-5*, which encodes a Delta-9 desaturase that specifically desaturates palmitic acid (PA;16:0) to palmitoleic acid (POA; 16:1(n-7)). In the worm, POA is readily elongated to cis-vaccenic acid (cVA; 18:1(n-7)), the most abundant fatty acid in phospholipids and neutral lipids in *C*. *elegans*. Therefore, we examined whether cVA is also protective in ferroptosis. To test this, we used a genetic approach to parse OA from cVA by using the double mutant *fat-6;fat-7* animals, that lack OA but are enriched for cVA (Fig 5A) [51]. Upon placing *fat-6;fat-7* on DGLA supplemented media, we found that they were highly resistant to DGLA-induced germ cell ferroptosis (Fig 5B), suggesting that, much like in *fat-2*, endogenous cVA are also protective from ferroptosis as well.

To extend these genetic findings, we tested whether dietary cVA would be protective in DGLA-induced ferroptosis. Upon repeating our previous experiment with 0.1mM OA and varying doses of DGLA, both WT and *ads-1* worms were protected from DGLA-induced ferroptosis (Fig 5C). When worms were supplemented with 0.1mM cVA in conjunction with varied doses of DGLA, we found that WT and *ads-1* worms both were strongly rescued and had reduced sterility, similar to supplementation with OA (Fig 5C). Because MUFAs, especially OA, were shown to play a role in lifespan [52], and were observed to be protective from ferroptosis in mammalian systems through reducing lipid peroxidation [12], we asked if the protection conferred by OA in the absence of ether lipids would lead to reduced lipid peroxidation. We measured MDA in worms treated with DGLA, OA, VA, or DGLA combined with OA and VA, and found significant reduction in lipid peroxidation end products when OA and VA are included in the dietary mixture (Fig 5D). Overall, these data show that dietary C18-MUFAs powerfully suppress germ cell ferroptosis by reducing lipid peroxidation products, which are key molecular executioners of ferroptotic cell death.

## Discussion

We previously found that depletion of ether lipids led to increased sensitivity to DGLA-induced ferroptosis in *C*. *elegans* [13], strongly suggesting that they are protective against this process. Because the vinyl-ether double bond in the subset of ether lipids called plasmalogens was proposed to act as an endogenous antioxidant that can “trap” lipid peroxides, we tested whether plasmalogens specifically were involved in the protection against DGLA-induced ferroptosis. Surprisingly, when we eliminated the plasmalogen subclass of ether lipids by a knockout mutation in the *tmem-189* gene, we saw no effect on the severity of germ cell death induced by DGLA. In addition, in an assay of whole-body oxidative stress induced by the toxicant TBHP, we found that the *tmem-189* mutant strain showed similar survival to wild type, indicating that plasmalogens are not acting as antioxidants to protect cells from DGLA- or peroxide-induced oxidative stress. In agreement with this, a recent study found that CRISPR-Cas9 knockout of *TMEM189* in mammalian cancer cells also did not alter ferroptotic sensitivity [19]. In contrast, another study showed that deleting *TMEM189* led to increased ferroptosis sensitization, while overexpression led to increased resistance to ferroptosis, although the protection was actually due to plasmalogens degrading fatty acyl CoA reductase (FAR1), essentially acting as a feedback inhibitor of ether lipid biosynthesis [20].

Thus, both studies showed that ether lipids themselves are pro-ferroptotic in mammalian systems, while another study showed little effect of ether lipid deficiency in mammalian cells [37]. In contrast, ether lipid biosynthesis mutants clearly showed increased sensitivity to ferroptosis in *C*. *elegans* germ cells. One explanation for this disparity may be attributed to the compositional differences of ether lipids and plasmalogens in *C*. *elegans* and mammalian cells. In mammals, plasmalogens make up a significant portion of phospholipids and are broadly distributed in different tissues, with the phosphatidylethanolamine (PE) fraction containing greater than 50% PE-plasmalogens in human brain, heart, and immune cells such as neutrophils, macrophages and lymphocytes, and the phosphatidylcholine (PC) fraction containing approximately 40% plasmalogens in human heart tissue and neutrophils [21, 53–57]. In addition, mammals have an abundance of AA in the sn-2 position where, for example, PE-plasmalogens contain approximately 75% AA in neutrophils and cardiac tissue [57, 58]. *C*. *elegans*, on the other hand, has a smaller cadre of ether lipids. The PE lipids consist of 20% alkyl (o-linked) ether lipids and 7% plasmalogen (p-linked) ether lipids. Unlike mammals, ether lipids are undetectable in PC [36, 59]. Additionally, *C*. *elegans* alkyl ether lipids are composed of approximately 60% MUFAs and saturated fats, and about 40% PUFAs in the sn-2; while in plasmalogen classes there are equal amounts of both MUFAs and PUFAs in the sn-2 position [36, 59, 60]. Of the PUFAs, only 3% and 12% are AA in the sn-2 in total PE-alkyl ether and PE-plasmalogen groups, respectively [36]. These data point to the species-specific differences in both mammals and *C*. *elegans*, as well as context-dependent requirements for lipid metabolism genes [37], and suggest that that PUFA composition in the sn-2 of either alkyl ether lipids or plasmalogens might drive their pro-ferroptotic role in mammals.

**Fig 6:**
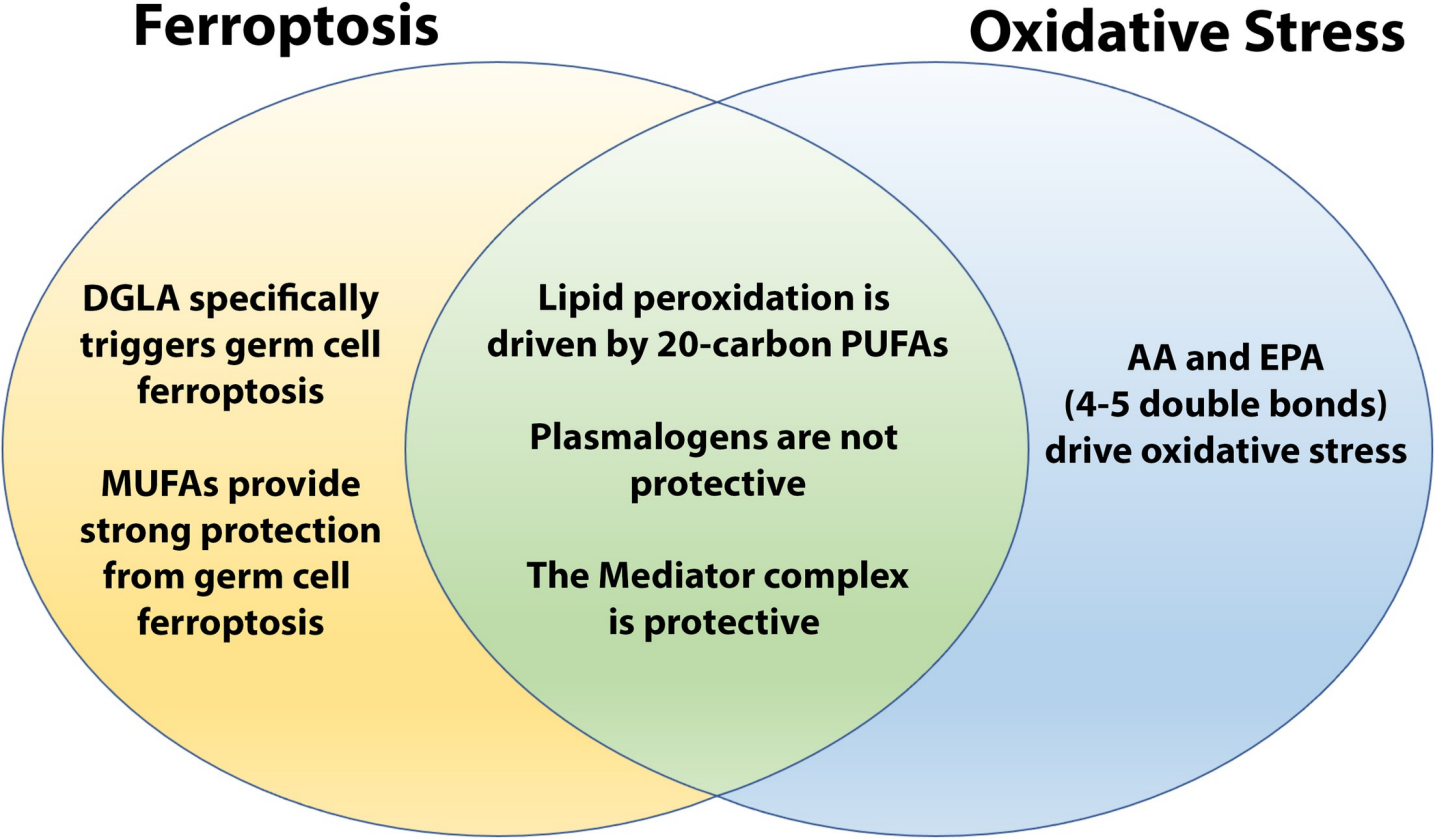
Similarities and differences between DGLA-induced ferroptosis and TBHP-induced oxidative stress. Ether lipid deficiency in *C*. *elegans* results in increased sensitivity to 20-carbon polyunsaturated fatty acids (PUFAs) through germ cell ferroptosis specifically induced by dietary and endogenous dihomo-gamma-linolenic acid (DGLA; 20:3(n-6)) and to oxidative stress that requires the highly unsaturated PUFAs, arachidonic acid (AA; 20:4(n-6)) and eicosapentaenoic acid (EPA; 20:5(n-3)). In both cases, the Mediator complex is protective while plasmalogens do not play an active role in protection as an endogenous antioxidant. Monounsaturated fatty acids (MUFAs) strongly protect worms from DGLA-induced ferroptosis.

As we probed further into the roles of ether lipids in the protection from DGLA-induced ferroptosis, we examined endogenous and dietary roles of specific MUFAs and PUFAs. This allowed us to explore the differences between DGLA-induced ferroptosis and peroxide-induced whole-body oxidative stress. We found that both treatments promote the generation of lipid peroxides, which accumulate to higher levels in ether-lipid deficient worms compared to WT. Moreover, in *tmem-189* mutants, these lipid peroxidation end products do not accumulate any more than WT, precluding plasmalogens as endogenous antioxidants as mentioned. However, in the absence of endogenously synthesized C20 PUFAs, the presence of ether lipids was no longer required for protection. Indeed, we found that *ads-1;fat-3* mutants were resistant to both peroxide-induced oxidative stress (this study) and DGLA-induced ferroptosis [13]. However, in the absence of ether lipids, increasing endogenous DGLA through genetic ablation of the omega-3 desaturase FAT-1 or the Delta-5 desaturase FAT-4, led to increased sensitivity to DGLA-induced ferroptosis. However, for peroxide-induced oxidative stress, we found opposite results for the two double mutant strains. The *ads-1;fat-1* and the *fat-1* worms, which accumulate excess DGLA and AA, were sensitive to TBHP, however, the *ads-1;fat-4* worms, along with the *fat-4* worms, were resistant to TBHP, in agreement with another report that *fat-4* RNAi worms survived longer in the context of oxidative stress [61]. Thus, our genetic and supplementation experiments revealed that an important distinction between germ cell ferroptosis and peroxide-induced whole-body oxidative stress is that the highly unsaturated fatty acids AA and EPA were more detrimental in whole-body oxidative stress but did not trigger ferroptotic germ cell death (Fig 6).

In the context of ferroptosis, MUFAs, particularly OA, have been shown to promote ferroptosis resistance in cancer cell lines, *C*. *elegans*, and in a mouse model of cancer [12, 13, 62]. Here we showed that both OA and cVA led to robust protection from DGLA-induced ferroptosis, and this protection was mechanistically conferred through reduced lipid peroxidation end products. We also found that the mediator complex component, MDT-15, involved in both fatty acid desaturation and stress response provided protection from both DGLA-induced ferroptotic germ cell death and in peroxide-induced oxidative stress. Furthermore, when *mdt-15(gof)* was crossed into *ads-1* mutants, the *ads-1;mdt-15(gof)* double mutants had a stress response similar to wild type in both stress modalities, correlating with an increase in transcription of *fat-5* and *fat-7*. Previously, Han et al showed that exogenous treatment of OA, POA, and cVA promote life and health span extension when studying epigenetic modifiers of H3K4me3 in *C*. *elegans* [52]. Knockdown of the COMPASS component *ash-2*/*ASH2L* leads to an increase in all MUFAs (POA, OA, and cVA) and this involved MDT-15, as knockdown of both *mdt-15* and *ash-2* abolished the increase in MUFAs from *ash-2* knockdown [52]. Moreover, another study showed that frailty from aging in *C*. *elegans* is correlated with increased iron and increased MDA levelsresulting in intestinal cell death by ferroptosis. Treatments that inhibited ferroptosis led to increased life and health span [63]. We show that MUFAs are strongly protective in the context of ferroptosis of germ cells and reproduction, and it would be interesting to determine whether MUFAs promote life and health span extension through the reduction of ferroptosis during aging.

Dietary MUFAs are important for optimal health and consumption of MUFAs has been associated with better cardiovascular health and reduced levels of diabetes [64–66]. Ferroptosis has been shown to occur in models of cardiovascular disease [67–70], renal disease [68, 71], and neurodegenerative diseases [1, 72–74] and ferroptosis-specific inhibitors were shown to protect in these studies. Our results suggest that dietary MUFAs act therapeutically to prevent or limit ferroptosis that occurs during these disease states.

## Materials and methods

### Worm strains and maintenance

All strains were maintained on NGM supplemented with OP50 and incubated at 20°C. The following strains were used in this study: N2, wild type; BX10 *ads-1(wa3)* [36]; BX24 *fat-1(wa9)*; BX30 *fat-3(wa22)*; BX17 *fat-4(wa14)*; BX52 *fat-1(wa9);fat-4(wa14)* [39]; XA7702 *mdt-15(ttm2182)* [75]; QC152 *mdt-15(et14)* was a gift from the Pilon lab, University of Gothenburg, Sweden; The strains BX295 *ads-1(wa3);fat-1(wa9)*; BX291 *ads-1(wa3);fat-3(wa22)*; BX290 *ads-1(wa3);fat-4(wa14);*and BX303 *ads-1;mdt-15(et14)* were generated by crossing *ads-1(wa3)* males to hermaphrodites. The F1 generation were allowed to self-cross, and the double mutants were identified in the F2 generation using GC/MS analysis for identification of the presence of homozygous alleles of *ads-1(wa3)*, *fat-3(wa22*), or *fat-1(wa9)* or PCR for identification of *mdt-15(et14)* and confirmation *of ads-1(wa3)* alleles (see below).

### Generation of the tmem-189 mutant

The *tmem-189(syb2649)* allele was made by SunyBiotech. It contains an in-frame 1128 bp deletion generated using CRISPR/CAS9 in the *tmem-189* gene on chromosome I. The deletion spans from just after the start codon and deletes exons 1, 2, 3, and part of exon 4. The resulting mutant strain was outcrossed 4x to wild type prior to use in these studies. To screen the mutants during the outcross, the primer sets for *tmem-189(syb2649)* were: FWD (5’-TCAAGTGGGAAAGCGTGTGA-3’), and REV (5’-CGCACGCCTAACCAAATCAT-3’). The wild type amplicon size was 2153bp, while the mutant amplicon 1025bp.

### Generation of the ads-1;mdt-15(gof) double mutant strain

Because *ads-1(wa3)* and *mdt-15(et14)* are both point mutants, we utilized tetra primer Amplification-Refractory Mutation System (ARMS)-PCR to generate the *ads-1;mdt-15(gof)* double mutant. ARMS-PCR measures allele-specific amplification through designing primers with mismatches in the 3’ end of the primer with a mismatch specific to the SNP of one allele and another mismatch 2 bases upstream from the 3’ end, and external flanking primers to produce allele-specific fragments that can be analyzed with PCR [76]. The primers used for screening *ads-1(wa3)* were as follows: mutant forward (5’- TGAGGTTTCGAACGACTTGT—3’); wild type reverse (5’- ATGAACCATGGTGTGCTAGG-3’); flank forward (5’-TGCTCCGTTCGTGGTCAGCAT-3’); flank reverse (5’-ACGTTGGGAGTGGTGTCCGA-3’). The primers used for screening *mdt-15(et14)* were as follows: wild type forward (5’-TCTTGCCTGAGCTGATGGTG-3’); mutant reverse (5’- GTGCCTCCAGATCCACAGCT-3’); flank forward (5’- GAACTGATGAAGGACCGGTTG-3’); flank reverse (5’- AGCCTGAGTTGGCGAGAAAC-3’). To screen for the double mutants, single worm PCR was performed, and half the proteinase K digestion underwent PCR amplification for either *ads-1(wa3)* or *mdt-15(et14)* ARMS-PCR primers and compared to controls. For *ads-1(wa3)* the flank fragment was 891bp, and the allele fragments were 395bp and 535bp for WT and mutant, respectively. For *mdt-15(et14)* the flank fragment was 962bp, and the allele fragments were 361bp and 661bp for WT and mutant, respectively.

### Peroxide-induced oxidative stress survival assays

Young adult day 1 worms were placed on freshly made NGM plates supplemented with 14.7mM tert-butyl-hydroperoxide (TBHP) before seeding with OP50. For TBHP plates, roughly 100–200 worms were scored every 1.5 hours as live or dead. Plates were incubated at 20°C for the duration of the assay. Survival curves and hazard ratios were plotted and analyzed with GraphPad Prism. P-values were calculated using log-rank (Mantel-Cox) tests.

### Measuring lipid peroxidation with TBARS assay

Approximately 1,250 synchronized young adult day 1 worms were washed off NGM plates into 15mL conical tubes and brought to 5mL M9 containing 15mM TBHP, or no treatment M9 control, and nutated for 60 minutes. Worms were washed twice with M9 and placed into 1.5mL Bioruptor Plus TPX microtubes (Diagenode, Denville, NJ). Supernatant was removed and the resulting pellet was frozen in liquid nitrogen and stored at -80°C until the day of the assay. Samples were lysed and homogenized using a Bioruptor sonicator bath (Diagenode, Denville, NJ) set on ‘High’ with 10 cycles of 30 sec pulsing and 30 sec pauses at 4°C. Samples were spun down at 20,000xg for 25 minutes at 4°C. Measurement of lipid peroxidation was performed using the Cayman Chemical thiobarbituric acid reactive substances assay (TBARS) (TCA method) assay kit (Ann Arbor, MI) per instructor’s manual and the microplate was read spectrophotometrically at 535nm on a Bio Tek Cytation 3 plate reader (Winooski, VT). Samples were normalized to protein using a Pierce BCA assay kit. Statistical analysis was performed with Student’s t-test where statistically significant results had a p<0.05.*Fatty acid supplementation*

NGM media was supplemented with Tergitol at final concentration of 0.1% and various doses of fatty acid salt (purchased from NuCheck Prep) dissolved in water before seeding with OP50 [77]. Worms were synchronized via alkaline hypochlorite treatment and L1 larvae were plated onto tergitol control or fatty acids plates at L1 stage and allowed to grow at 20°C to reach young adult stage. For the DGLA assay, worms were scored visually under light microscopy for absence or presence of germ cell and embryos (scored as sterile or fertile, respectively) and then collected for GC-MS. For whole-body oxidative stress assays, worms were individually picked from tergitol control or fatty acid supplemented plates onto the TBHP assay and scored for survival as mentioned earlier, and a portion of the population was kept for GC/MS analysis.

### Fatty acid analysis

Fatty acid composition was analyzed by gas chromatography/mass spectrometry (GC/MS) using the fatty acid methyl ester (FAME) method [51]. For each sample, roughly 400 worms were collected in water, allowed to settle, and most of the water removed before freezing the worm pellet. 2.5% sulfuric acid in methanol was added to frozen pellets and then incubated at 70°C for one hour in a glass tube. FAMEs were extracted in hexane and separated using an Agilent 7890 GC/5975C MS in scanning ion mode equipped with a SP-1380 column. Relative amounts of fatty acid methyl esters are reported.

### Isolation of RNA and Quantitative RT-PCR analysis

Total RNA was isolated from synchronized L4 worms that were harvested and then frozen in liquid nitrogen, and then stored at -80°C. RNA was prepared using TRIzol Reagent (Invitrogen, Cat# 15596018) and purified with RNeasy Mini Plus Kit (Qiagen, Cat# 74134) per manufacturer’s instructions. The RNA was then converted to cDNA using SuperScript IV Reverse Transcriptase kit (Invitrogen, Cat# 18090010). Real-time quantitative PCR assays were run on an Applied Biosystems 7300 Real-Time PCR System (Applied Biosystems, Foster City, CA) with the following cycling parameters: 3 minutes at 95°C, then 40 cycles of 95°C for 15 seconds and 60°C for 1 minute. Fluorescence data was collected at the 60°C step. Each sample was run in triplicate and normalized to the genes *cdc-42* and *Y45F10D*.*4*, and all gene differential expression was normalized to wild type. The RT-qPCR primer sequences used for this study were as follows: *fat-5*: FWD (5’-CGATTTGTACGAGGATCCGGTG-3’) and REV (5’-CAGTGGGAGACACTGTTGATGC-3’); *fat-6*: FWD (5’-TCTACCAGCTCATCTTCGAGGC-3’) and REV (5’-GATCACGAGCCCATTCGATGAC-3’); *fat-7*: FWD (5’-GGAAGGAGACAGCATTCATTGCG-3’) and REV (5’-GTCTTGTGGGAATGTGTGGTGG-3’); *gst-4*: FWD (5’-GATGCTCGTGCTCTTGCTG-3’) and REV (5’-CCGAATTGTTCTCCATCGAC-3’); *gst-6*: FWD (5’-TTTGGCAGTTGTTGAGGAG-3’) and REV (5’-TGGGTAATCTGGACGGTTTG-3’); and *sod-3*: FWD (5’-GCTGCAATCTACTGCTCGCACTGCTTCAAAGC-3’) and REV (5’-GGCAAATCTCTCGCTGATATTCTTCCAGTTGGC-3’). The RT-qPCR primer sequences for the genes used for normalization were: *cdc-42*: FWD (5’-CTGCTGGACAGGAAGATTACG-3’) and REV (5’-CTCGGACATTCTCGAATGAAG-3’); *Y45F10D*.*4*: FWD (5’-GTCGCTTCAAATCAGTTCAGC-3’) and REV (5’-GTTCTTGTCAAGTGATCCGACA-3’). Statistical analysis was performed with Student’s t-test where statistically significant results had a p<0.05.

## Supporting information

S1 Table Fatty acid composition, sterility assays, and fatty acid uptake determined by gas chromatography/ mass spectrometry for Figs 1–5. (XLSX)

S2 Table Statistical analyses for assays shown in Figs 1–5. (XLSX)
